# Prediction of lateral lymph node metastasis with short diameter less than 8 mm in papillary thyroid carcinoma based on radiomics

**DOI:** 10.1186/s40644-024-00803-7

**Published:** 2024-11-15

**Authors:** Yan Wang, Shuangqingyue Zhang, Minghui Zhang, Gaosen Zhang, Zhiguang Chen, Xuemei Wang, Ziyi Yang, Zijun Yu, He Ma, Zhihong Wang, Liang Sang

**Affiliations:** 1 Department of Ultrasonography, The First Affiliated Hospital of China Medical University, Shenyang, Liaoning China; 2 School of Medical and Bioengineering Information, Northeastern University, Shenyang, Liaoning China; 3 Department of Thyroid Surgery, The First Affiliated Hospital of China Medical University, Shenyang, Liaoning China

**Keywords:** Radiomics, Ultrasound, Ensemble learning, Papillary thyroid carcinoma, Lateral cervical small lymph node

## Abstract

**Objective:**

The aim of this study was to establish an ensemble learning model based on clinicopathological parameter and ultrasound radomics for assessing the risk of lateral cervical lymph node with short diameter less than 8 mm (small lymph nodes were used instead) metastasis in patients with papillary thyroid cancer (PTC), thereby guiding the selection of surgical methods.

**Methods:**

This retrospective analysis was conducted on 454 patients diagnosed with papillary thyroid carcinoma who underwent total thyroidectomy and lateral neck lymph node dissection or lymph node intraoperative frozen section biopsy at the First Hospital of China Medical University between January 2015 and April 2022. In a ratio of 8:2, 362(80%) patients were assigned to the training set and 92(20%) patients were assigned to the test set. Clinical pathological features and radomics features related to ultrasound imaging were extracted, followed by feature selection using recursive feature elimination (RFE). Based on distinct feature sets, we constructed ensemble learning models comprising random forest (RF), extreme gradient boosting (XGBoost), categorical boosting (CatBoost), gradient boosting decision tree (GBDT), and light gradient boosting machine (Lightgbm) to develop clinical models, radiomics models, and clinical-radiomic models. Through the comparison of performance metrics such as area under curve (AUC), accuracy (ACC), specificity (SPE), precision (PRE), recall rate, F1 score, mean squared error (MSE) etc., we identified the optimal model and visualized its results using shapley additive exPlanations (SHAP).

**Results:**

In this study, a total of 454 patients were included, among whom 342 PTC patients had small lymph node metastasis in the lateral neck region, while 112 did not have any metastasis. A total of 1035 features were initially considered for inclusion in this study, which were then narrowed down to 10 clinical features, 8 radiomics features, and 17 combined clinical-omics features. Based on these three feature sets, a total of fifteen ensemble learning models were established. In the test set, RF model in the clinical model is outperforms other models (AUC = 0.72, F1 = 0.75, Jaccard = 0.60 and Recall = 0.84), while CatBoost model in the radiomics model is superior to other models (AUC = 0.91, BA = 0.83 and SPE = 0.76). Among the clinical-radiomic models, Catboost exhibits optimal performance (AUC = 0.93, ACC = 0.88, BA = 0.87, F1 = 0.91, SPE = 0.83, PRE = 0.88, Jaccard = 0.83 and Recall = 0.92). Using the SHAP algorithm to visualize the operation process of the clinical-omics CatBoost model, we found that clinical omics features such as central lymph node metastasis (CLNM), Origin_Shape_Sphericity (o_shap_sphericity), LoG-sigma3_first order_ Skewness (log-3_fo_skewness), wavelet-HH_first order_Skewness (w-HH_fo_skewness) and wavelet-HH_first order_Skewness (sqr_gldm_DNUN) had the greatest impact on predicting the presence of lateral cervical small lymph node metastasis in PTC patients.

**Conclusions:**

(1) In this study, among the ensemble learning models established based on clinicopathological features and radiomics features for predicting PTC lateral small lymph node metastasis, the clinical-radiomic CatBoost model has the best performance. (2) SHAP can visualize how the clinical and radiomics features affect the results and realize the interpretation of the model. (3) The combined CatBoost model can improve the diagnostic accuracy of suspicious lymph nodes with short diameter < 8 mm that are difficult to obtain accurate puncture results. The combined application of radiomics features is more accurate and reasonable than the prediction of clinical data alone, which helps to accurately evaluate the surgical scope and provide support for individual clinical decision making.

**Supplementary Information:**

The online version contains supplementary material available at 10.1186/s40644-024-00803-7.

## Introduction

Thyroid cancer is the most common endocrine malignancy, among which differentiated thyroid cancer (DTC) accounts for about 95%, and papillary thyroid cancer (PTC) is the most common, accounting for about 90% of adult thyroid cancer [1]. The prevalence of lateral lymph node metastasis (LLNM) in papillary thyroid carcinoma (PTC) patients has been reported to range from 21 to 63% based on scientific studies [2, 3]. Lymph node metastasis is a major risk factor for PTC recurrence [4], and when recurrence occurs, it may increase the risk of second surgery and increase the probability of postoperative complications [5]. Therefore, effective preoperative evaluation can make reasonable inferences and predictions for thyroid lateral lymph node metastasis (LLNM). It is of great significance for the selection of PTC staging and treatment [6].

Ultrasound is widely acknowledged as the preferred imaging modality for thyroid diseases [7], enabling effective evaluation of the status of lateral neck lymph nodes with a sensitivity ranging from 60 to 80%. However, it should be noted that the diagnostic accuracy of ultrasonography is significantly influenced by the expertise and experience of the operator [8, 9]. The indications for lateral neck lymph node dissection include ultrasound-guided lymph node puncture biopsy or intraoperative frozen biopsy, both of which require pathologically confirmed metastasis [10, 11]. According to the American Thyroid Association( ATA) in 2015, fine needle aspiration(FNA) is not recommended for lymph nodes with a short diameter of < 8 mm [11]. The European Thyroid Association (ETA) pointed out that lymph node diameter greater than 5 mm is considered to be a predictor of malignancy. Similarly, Xia et al. proposed that ultrasound-guided FNA has high accuracy in diagnosing lateral lymph node metastases with a diameter smaller than 6 mm [12, 13]. Clinically, machine learning models based on ultrasonographic features have been applied to evaluate central or lateral cervical lymph node metastasis of PTC, and have high diagnostic efficacy [14, 15]. However, there is no reliable machine learning model to evaluate the lateral cervical small lymph nodes [16](short diameter < 8 mm).

Compared with a single machine learning model, ensemble learning model can combine the advantages of multiple classifiers to make the prediction results more accurate. The shapley additive explanation (SHAP), proposed by Lundberg and Lee in 2017, is an additive feature attribution method that introduces SHAP values as a unified measure of feature importance, realizing local and global visualization, and improving the explainability of the model [17–19]. SHAP has been successfully applied to predict sentinel lymph node metastasis of breast cancer and distant metastasis of osteosarcoma [20, 21]. The purpose of this study was to establish ensemble learning models based on ultrasound image radiomics to predict the metastasis of lateral cervical small lymph nodes of PTC, and to visualize the prediction results of the ensemble learning model using the SHAP algorithm. This provides guidance for the management of suspicious lymph nodes that are difficult to obtain accurate puncture results.

## Material and method

### Population

**Fig. 1:**
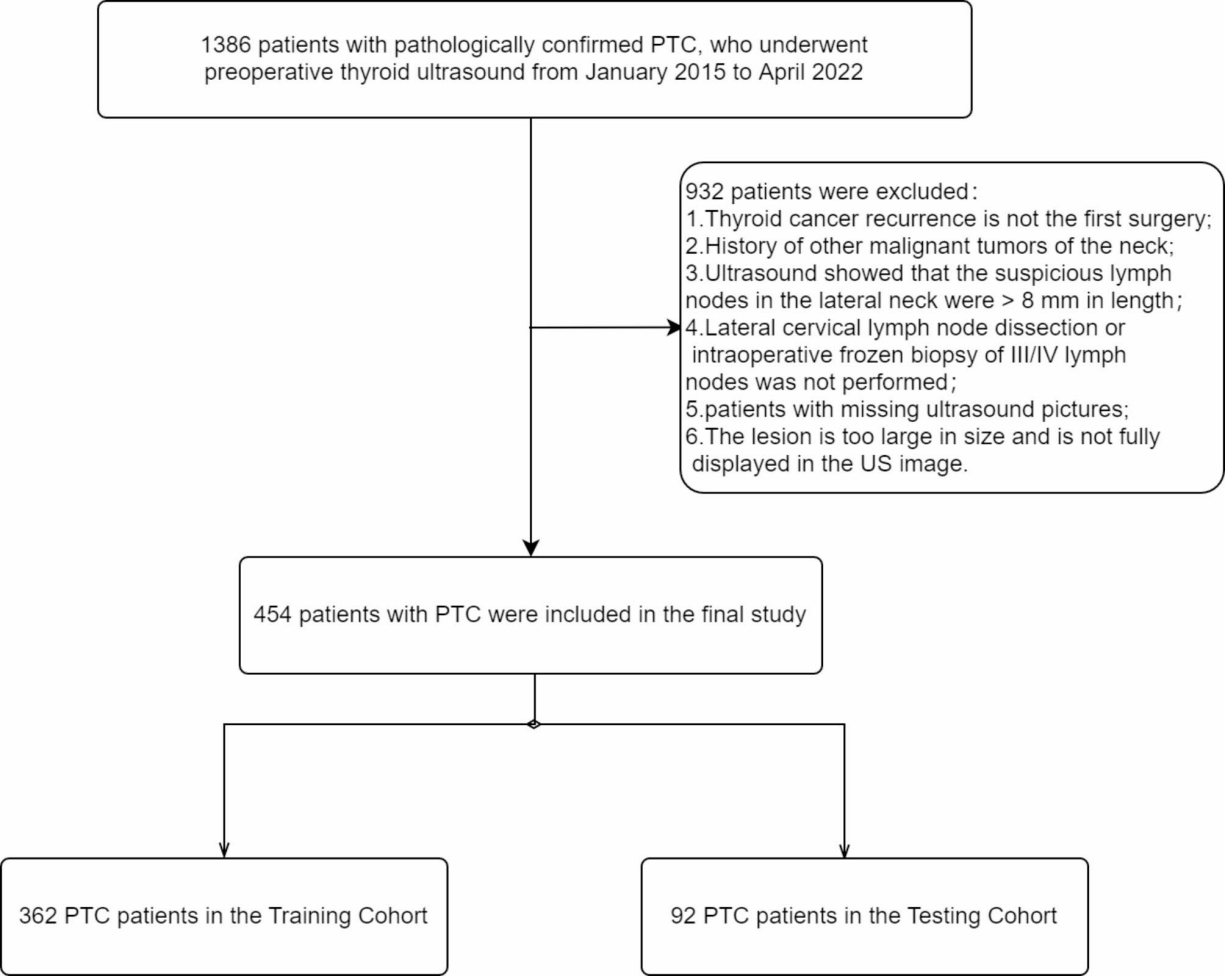
Study flow chart

This study follows the Declaration of Helsinki and was approved by the Medical Science Research Ethics Committee of the First Affiliated Hospital of China Medical University (AF-SOP-07-1.2-01). The requirement for written informed consent was waived due to the retrospective nature of this study. A retrospective analysis was made of 1386 patients who underwent thyroidectomy, lateral cervical lymph node dissection or intraoperative frozen biopsy in the Department of Thyroid Surgery, the First Affiliated Hospital of China Medical university from January 2015 to April 2022. Inclusion criteria: (1) patients had complete clinical data, and ultrasound examinations of thyroid and cervical lymph nodes were performed within one week before surgery; (2) the initial thyroid surgery should be accompanied by lateral neck lymph node dissection or intraoperative frozen biopsy of III/IV lymph nodes; (3) postoperative histopathological results confirmed PTC; (4) ultrasonography revealed that the short diameter of suspicious lymph node(SLN) in the lateral neck was less than 8 mm. SLN were defined as those exhibiting at least one of the following five ultrasonic features: round shape, focal strong echo, cystic degeneration, microcalcification, and peripheral blood vessels [11]. Exclusion criteria: (1) a medical history of prior cervical radiation exposure or treatment; (2) history of previous malignancies in the cervical region; (3) absence of conventional ultrasound images; (4) the US image failed to fully depict the lesion due to its considerable size. Out of these, a total of 932 patients were excluded from the study, while 454 patients were included in the final analysis. Out of these, a total of 932 patients were excluded from the study, while 454 patients were included in the final analysis. Among them, 362 patients were randomly assigned to the training set, and the remaining 92 patients were allocated to the test set (Fig. 1).

### Instrument

EPIQ7 (linear array probe = 5–12 MHz; Philips, Amsterdam, Netherlands), Preirus (linear array probe = 5–12 MHz; Hitachi, Tokyo, Japan) and AixPlorer (linear array probe = 4–15 MHz; Supersonic, France).

### Ultrasonic image characteristics and clinicopathological parameters

All participants underwent preoperative ultrasound examination within one week prior to the surgery. Then, the lesions were evaluated by ultrasound by physicians A and B with more than 10 years of experience in ultrasound examination. The clinical characteristics of the patients were retrospectively analyzed, encompassing demographic information, ultrasonic features of the primary thyroid lesion, and postoperative pathological findings. The basic information of patients includs: sex and age. Ultrasound features includes: tumor location (upper left, middle left, lower left, upper right, middle right or lower right); diameter; texture(even or uneven); echogenicity (hyperechoic, isoechoic, hypoechoic, very hypoechoic or anechoic); composition (solid, solid dominate, cystic, cystic dominate or spongy); boundary(clear or dim); orientation(wider-than-tall or taller-than-wide); posterior features(enhancement, attenuation, unaltered, mixed changes); mulifocality; halo; microcalcification; benign nodules and SLN. Pathology information includes: prelaryngeal lymph node (PLN), central lymph node (CLN), hashimoto’s thyroiditis (HT), capsular invasion.

### Division of areas of interest

A physician with a decade of expertise in thyroid ultrasound meticulously delineates regions of interest (ROI) on ultrasound images for the purpose of identifying thyroid lesions.

### Data preprocessing and feature screening

The ultrasonic image data is subjected to mixup image amplification, followed by the application of various filters on the amplified image data. These filters include laplacian of gaussian (LoG, σ = 1, 3), wavelet, square, square root, exponential, and gradient. The utilization of pyradiomic (v3.0.1, https://github.com/Radiomics/pyradiomics) in ultrasound images facilitates the extraction of ROI characteristics pertaining to radiomics analysis. Firstly, the non-counting features undergo max-min normalization, followed by a subsequent screening of these features through recursive feature elimination (RFE).

### Model construction

Random forest (RF), extreme gradient boosting (XGBoost), categorical boosting (CatBoost), gradient boosting decision tree (GBDT), and light gradient boosting machine (Lightgbm). Classifiers were utilized to construct clinical models, radiomics models, and clinical-radiomic combined models based on different feature sets obtained after pretreatment. In the process of model construction, a 5-fold cross-validation is performed on the training set by randomly partitioning it based on patients, and subsequently evaluating the final performance of this predictive model using the test set. Data analysis incorporates five machine learning algorithms implemented in Python (version 3.10), namely xgboost (https://github.com/dmlc/xgboost/), lightgbm (https://github.com/microsoft/LightGBM/), catboost (https://github.com/catboost/), as well as RF and GBDT from Scikit-learn (https://scikit-learn.org/stable/).

### Selection and validation of Ensemble Learning models

The performance of the model was assessed on both training and validation datasets using established clinical statistical measures, including area under the curve (AUC), accuracy, specificity, precision, sensitivity, F1 score, and mean squared error(MSE). The diagnostic performance was compared, and the best-performing model set on the test dataset was identified and evaluated to select the classifier with the optimal predictive performance.

### SHAP visualization

**Fig. 2:**
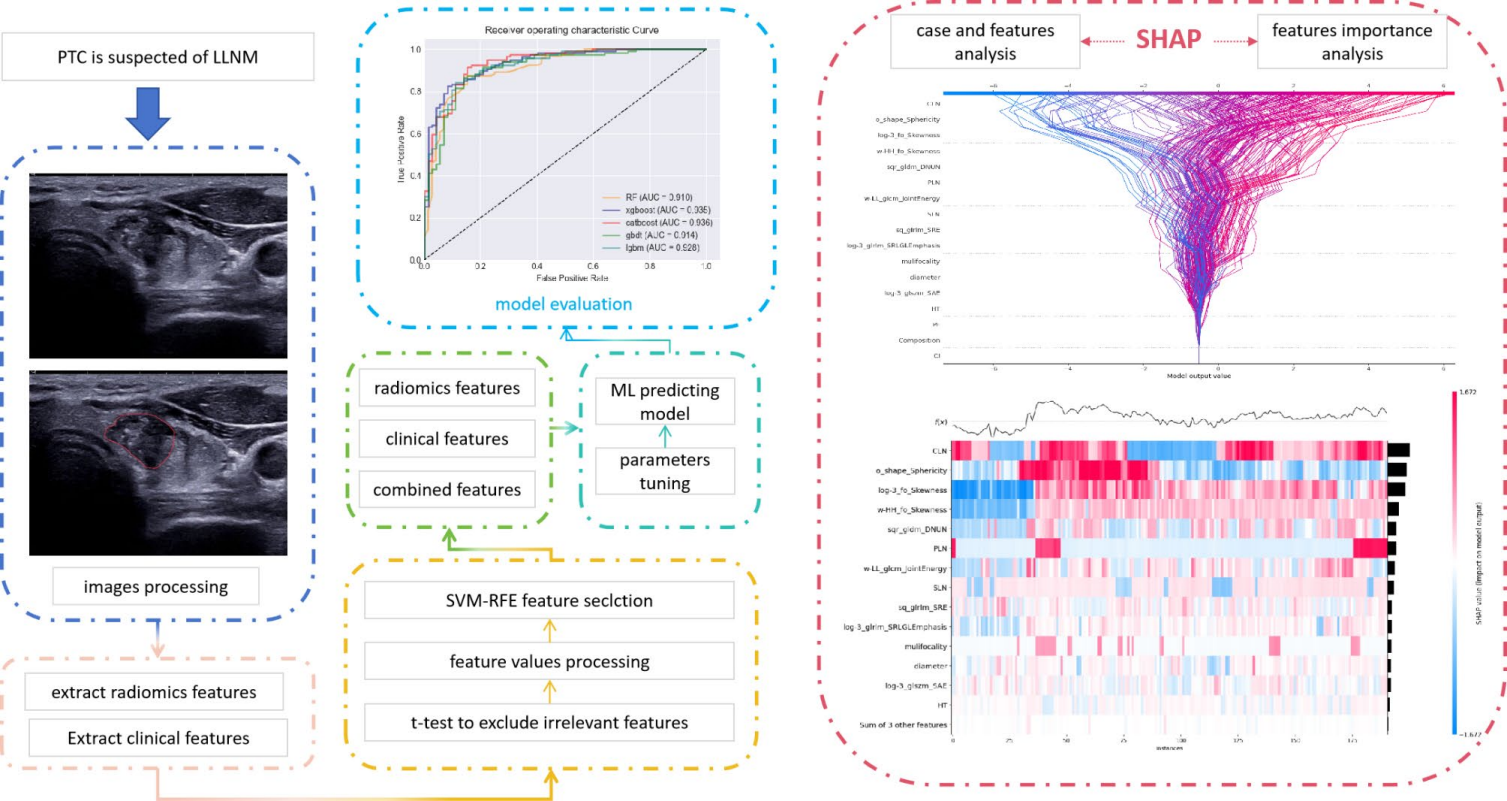
The flow diagram of the research

The SHAP Python framework (version 0.41.0) was utilized for visualizing the optimal model, employing SHAP (https://github.com/slundberg/shap). Figure 2 illustrates the process of constructing a model based on clinical and radiomics features, presented using SHAP.

### Statistical analysis

SPSS (v. 27.01; IBM Corp., Armonk, NY, USA) statistical software was used. The independent samples t-test is employed for continuous variables that adhere to a normal distribution, while the wilcoxon rank-sum test is utilized for those that do not. For categorical variables, both the Pearson chi-square test and Fisher’s exact probability test are applied. *P* < 0.05 indicates statistical significance of the observed differences.

## Result

### Clinical features of papillary thyroid carcinoma

This study included a total of 454 patients diagnosed with PTC, among whom 342 cases exhibited small lymph node metastasis in the lateral neck region, while 112 cases showed no evidence of metastasis. The patients were randomly divided into training and testing sets in an 8:2 ratio (Fig. 1). A total of 362 patients (80%) were assigned to the training set, comprising of 90 males and 272 females, with a median age of 37 years (range: 30–48 years). A total of 92 patients (20%) were allocated to the testing set, comprising 31 males and 61 females, with a median age of 36.5 years (range: 29.5–47 years). The mean tumor diameter was 1.59 ± 0.89 centimeters, as determined by postoperative pathology analysis. Preoperative ultrasound findings indicated SLN in 387 patients, and subsequent pathological examination confirmed CLNM in 309 patients, including 243 in the training set and 64 in the testing set. Additionally, concurrent Hashimoto’s thyroiditis was observed in 125 patients. For categorical variables, inter-observer agreement analysis was conducted for radiologists A and B, with K values > 0.8. All ultrasound feature evaluation results in this study are based on the assessments of radiologist B.

Table 1 presents a comprehensive inventory of the clinical characteristics associated with 454 patients diagnosed with PTC, encompassing both the training and testing cohorts. Significant differences (*P* < 0.05) were found in the training set between patient gender, age, multifocality, SLN, PLN and CLN in terms of the existence of lymph node metastases in the lateral neck. Age, diameter, posterior feature, and CLN all showed statistically significant variations in the testing set. (*p* < 0.05)

**Table 1:** Univariate analysis of lateral cervical micro-lymph node metastases of papillary thyroid carcinoma was concentrated in the training and testing sets

variables	Trating set (*N* = 362)			testing set(*N* = 92)		
	LLNM (-)	LLNM(+)	*P* value	LLNM (-)	LLNM (+)	*P* value
	*N* = 89	*N* = 273		*N* = 23	*N* = 69	
Sex			0.044			0.524
Male	15	75		9	22	
Female	74	198		14	47	
Age	45(34,54)	36(29,45)	< 0.001	42.35(30.06,54.64)	36.80(25.97,47.63)	0.043
Diameter	1.31(0.85,1.89)	1.43(1.05,2.0)	0.093	0.98(0.69,1.47)	1.3(1.01,1.76)	0.027
Location			0.078			0.551
Upper left	12	58		4	19	
Middle left	17	47		2	7	
Lowe left	16	26		5	10	
Upper right	15	64		4	17	
Middle right	10	38		6	8	
Lower right	19	40		2	8	
Mulifocality			0.04			0.088
No	85	240		22	53	
Yes	4	33		1	16	
Benign nodule			0.51			0.273
No	27	73		4	20	
Yes	62	200		19	49	
Boundary			0.059			0.487
Clear	61	214		16	53	
Dim	28	59		7	16	
Echogenicity			0.826			0.448
Hyperechoic/iso-echoic	3	14		2	3	
Hypoechoic	86	259		21	66	
Composition			0.507			0.163
Solid	84	263		20	66	
Solid dominate	5	9		3	3	
Cystic dominate	0	1		0	0	
Texture			0.241			1
Even	8	15		1	4	
Uneven	81	258		22	65	
Microcalcification			0.145			0.39
No	48	123		11	26	
Yes	41	150		12	43	
Posterior feature			0.534			0.047
Enhancement	0	0		2	0	
Attenuation	21	53		7	13	
Unaltered	63	209		13	54	
Mixed changes	5	11		1	2	
Orientation			0.363			0.803
Wider-than-tall	57	189		15	43	
Taller-than-wide	32	84		8	26	
Halo			0.131			0.068
No	75	246		18	65	
Yes	14	27		5	4	
SLN			< 0.001			0.863
No	25	29		4	9	
Yes	64	244		19	60	
PLN			<0.001			0.112
No	89	208		22	54	
Yes	0	65		1	15	
CLNM			< 0.001			0.009
Yes	22	221		11	53	
No	67	52		12	16	
Number of CLNM	0(0,0.5)	3(1,5)	<0.0001	0(0,3)	2(1,5)	0.013
HT			0.036			0.422
No	57	206		18	48	
Yes	32	67		5	21	
Capsular invasion			0.111			0.284
No	81	230		22	58	
Yes	8	43		1	11	

LLNM, lateral lymph node metastasis; SLN, suspicious lymph nodes; PLN, prelaryngeal lymph node; CLN, central lymph node

### Evaluation of model performance and clinical utility

**Fig. 3:**
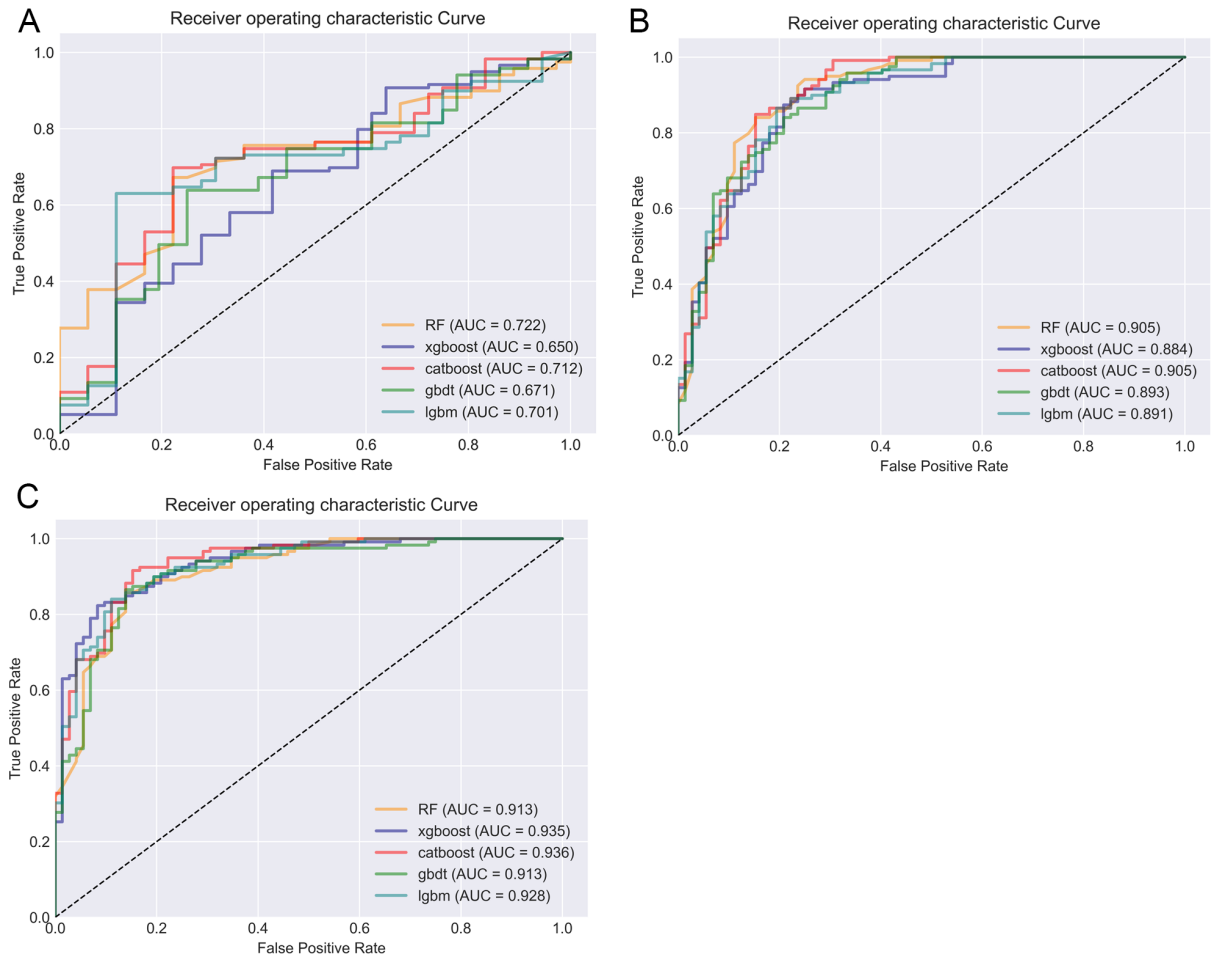
(**A**) A ROC curve for clinical model. (**B**) B ROC curve of radiomics model set. (**C**) ROC curve of clinico-radiomics model

**Fig. 4:**
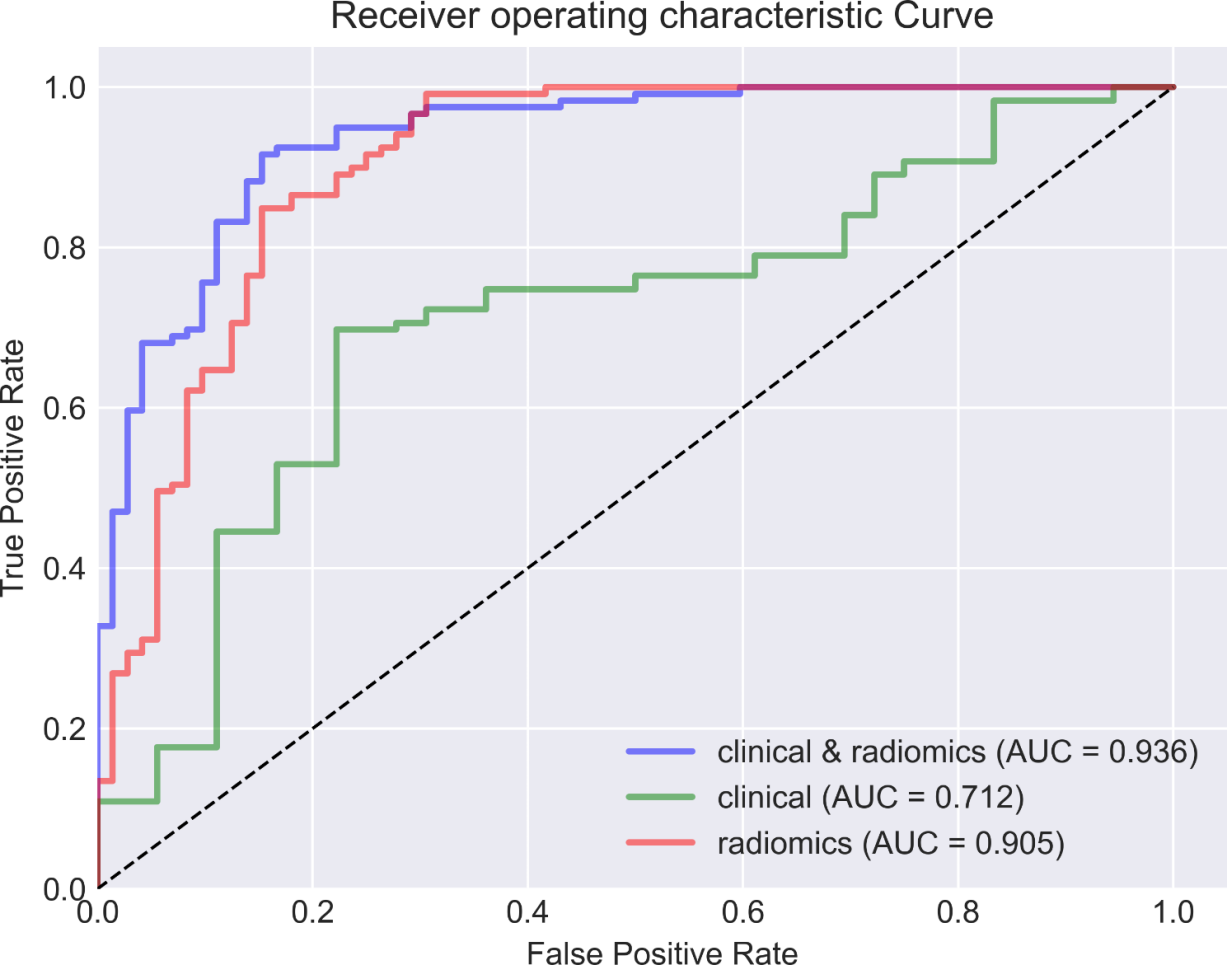
ROC curve based on CatBoost model of three data

**Fig. 5:**
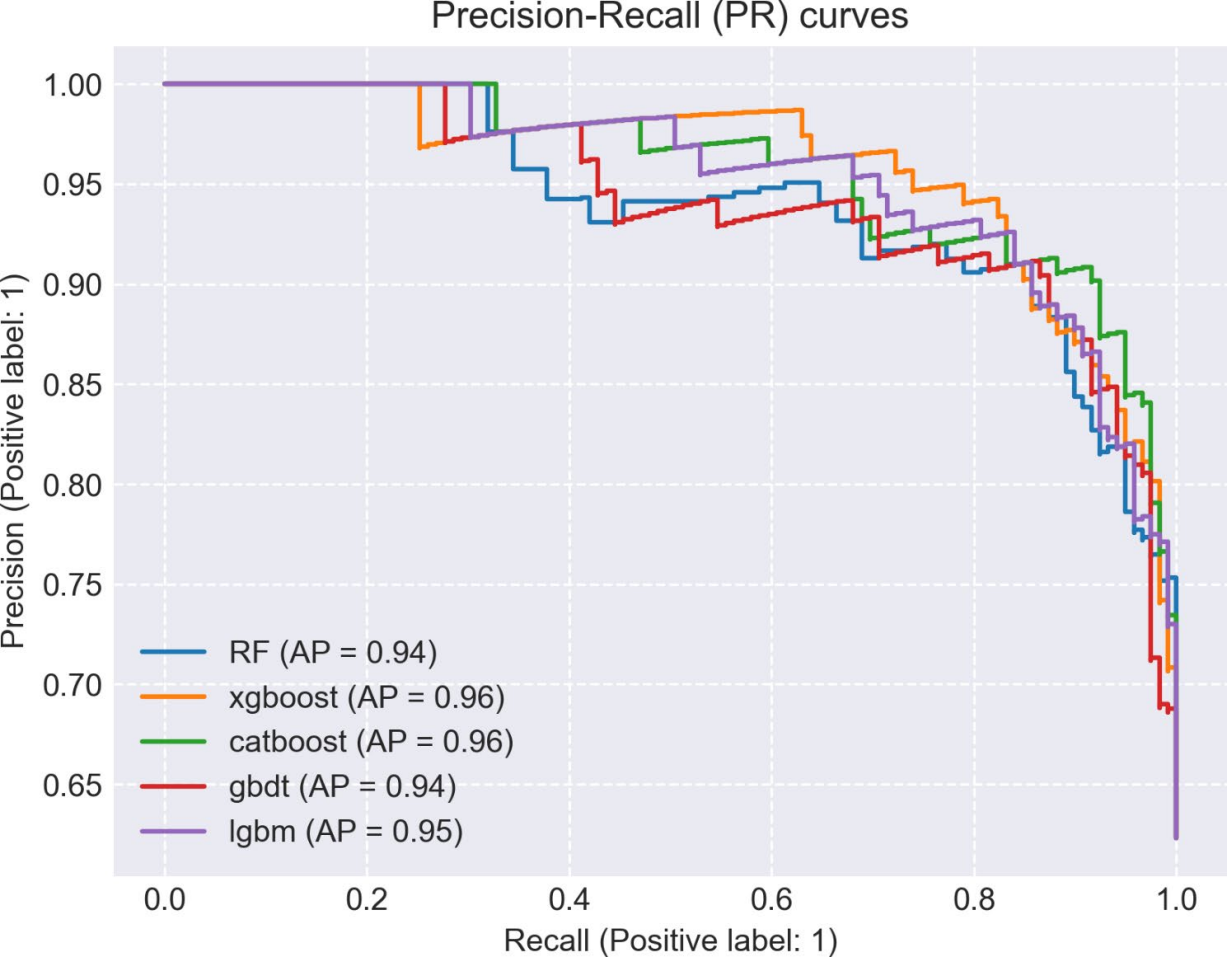
Precision-recall characteristics of clinico-radiomics model

**Fig. 6:**
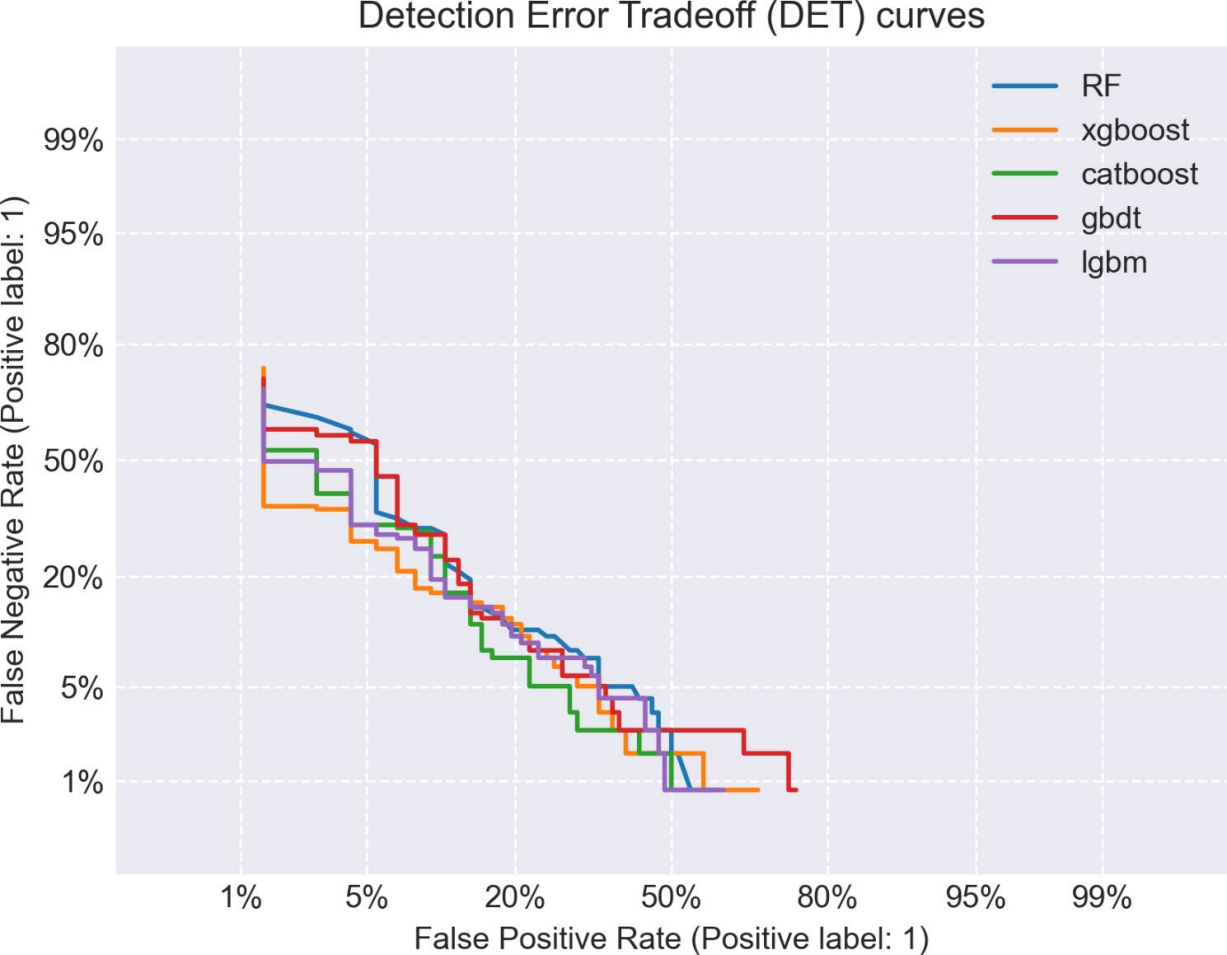
The calibration curve of the clinical-radiomic model of CatBoost

**Fig. 7:**
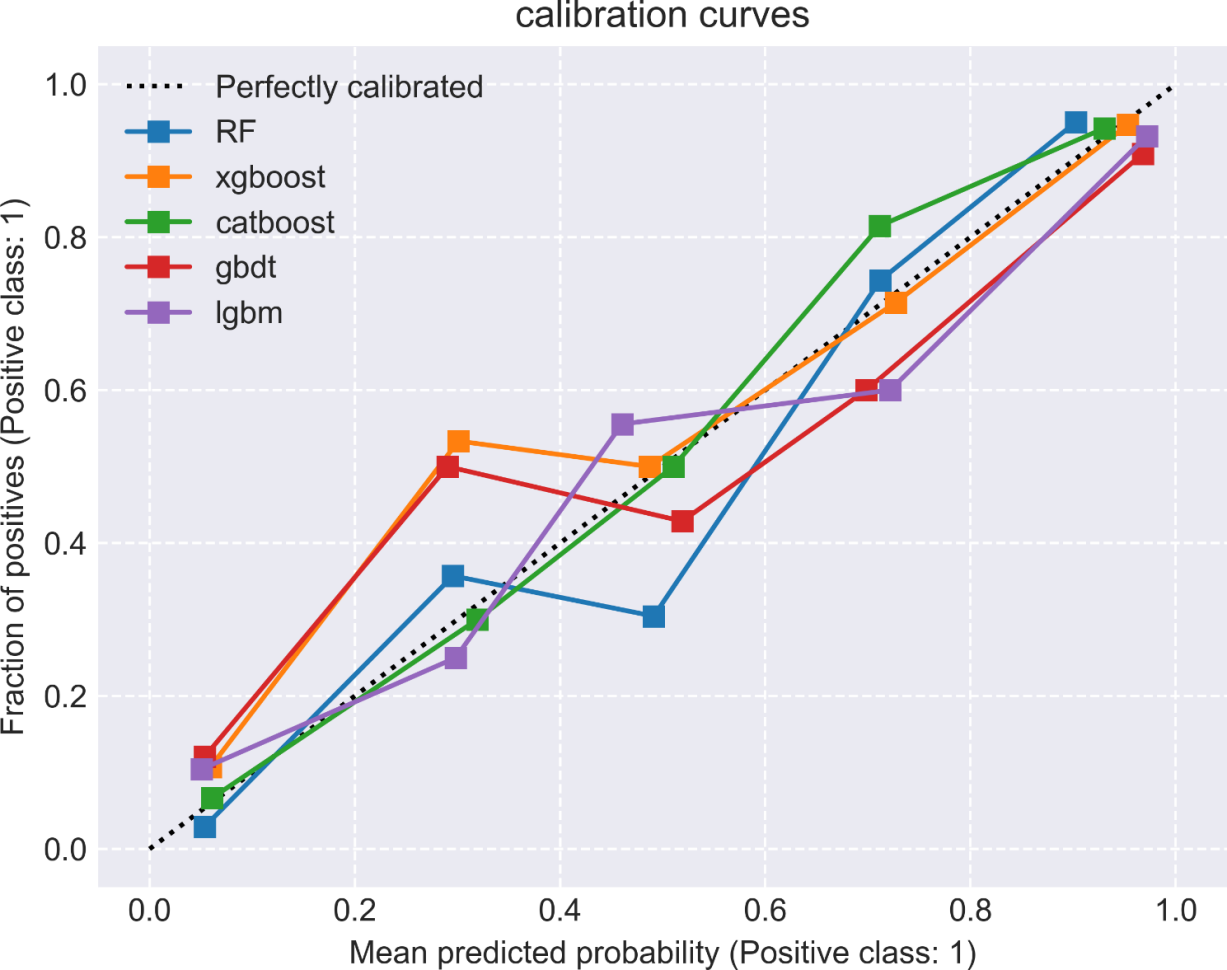
Detection error trade-off curves of clinico-radiomics model

The study encompassed a total of 1035 features, and feature selection was conducted using RFE, resulting in the identification of 10 individual clinical features, 8 individual radiomics features, and 17 combined clinical-radiomic features (supplementary table). Based on this rationale, a total of 15 models were constructed, encompassing RF, XGBoost, CatBoost, GBDT and Lightgbm models (Table 2). By comparing the AUC values of different models, it can be observed that the RF model outperforms other models in terms of clinical model (Fig. 3A), with an AUC = 0.72, f1 = 0.75, Jac = 0.60, and recall = 0.84. In radiomics model, the CatBoost model demonstrated superior performance compared to other models (Fig. 3B), achieving a bac = 0.83, spe = 0.76, and AUC = 0.91. Among all the models, the clinical-radiomic CatBoost model demonstrates superior performance in predicting lateral cervical small lymph node metastasis in PTC (Fig. 3C), exhibiting an AUC = 0.93, ACC = 0.88, Ba = 0.87, f1 = 0.91, spe = 0.83, pre = 0.88, Jac = 0.83 and recall = 0.92; additionally, it achieves the lowest MSE at 0.33 (Table 2). Compared to individual clinical or radiomics CatBoost models, the combined model exhibits superior predictive performance with the highest area under the ROC curve (Fig. 4), while also demonstrating optimal precision-recall characteristics (Fig. 5). Compared to individual clinical or radiomics CatBoost models, the combined model exhibits superior predictive performance with the highest area under the ROC curve (Fig. 4), while also demonstrating optimal precision-recall characteristics (Fig. 5). The calibration curve of the clinical-radiomic model of CatBoost exhibits remarkable consistency with bias-corrected predicted values (Fig. 6). The detection error trade-off (DET) curves reveal that all five datasets exhibit a concentration in the third quadrant, with the CatBoost model demonstrating the most favorable error rejection rate and error acceptance rate (Fig. 7).

**Table 2:** Predictive performance of ensemble learning models in training and test sets

Training set										Testing set								
Clinical model																		
	ACC	BA	F1	SPE	PRE	JAC	RECALL	MSE	AUC	ACC	BAC	F1	SPE	PRE	JAC	RECALL	MSE	AUC
RF	0.714	0.671	0.786	0.485	0.703	0.857	0.649	0.533	0.785	0.649	0.587	0.749	0.333	0.617	0.599	0.840	0.592	0.722
xgboost	0.728	0.696	0.791	0.552	0.716	0.841	0.655	0.520	0.774	0.607	0.570	0.696	0.417	0.574	0.534	0.723	0.627	0.650
catboost	0.758	0.743	0.801	0.693	0.741	0.793	0.669	0.491	0.810	0.675	0.652	0.742	0.556	0.653	0.589	0.748	0.570	0.712
gbdt	0.707	0.661	0.780	0.469	0.691	0.853	0.641	0.540	0.786	0.649	0.598	0.741	0.389	0.617	0.589	0.807	0.592	0.671
lgbm	0.716	0.676	0.784	0.506	0.704	0.846	0.646	0.532	0.805	0.633	0.596	0.718	0.444	0.603	0.560	0.748	0.605	0.701
Radiomics model																		
RF	0.847	0.819	0.882	0.697	0.857	0.941	0.789	0.390	0.879	0.859	0.829	0.893	0.708	0.869	0.807	0.950	0.376	0.905
xgboost	0.834	0.821	0.866	0.760	0.825	0.882	0.765	0.407	0.884	0.838	0.823	0.871	0.764	0.829	0.772	0.882	0.403	0.884
catboost	0.852	0.839	0.882	0.773	0.846	0.904	0.790	0.383	0.892	0.848	0.832	0.881	0.764	0.842	0.787	0.899	0.390	0.905
gbdt	0.854	0.833	0.885	0.739	0.855	0.927	0.794	0.382	0.884	0.832	0.808	0.871	0.708	0.830	0.771	0.908	0.409	0.893
lgbm	0.851	0.826	0.885	0.712	0.857	0.940	0.794	0.386	0.883	0.832	0.813	0.869	0.736	0.826	0.768	0.891	0.409	0.891
Clinico-radiomic model																		
RF	0.863	0.842	0.892	0.758	0.862	0.926	0.805	0.370	0.935	0.832	0.813	0.869	0.736	0.826	0.768	0.891	0.409	0.910
xgboost	0.880	0.867	0.904	0.814	0.876	0.920	0.824	0.346	0.944	0.848	0.845	0.876	0.833	0.837	0.779	0.857	0.390	0.935
catboost	0.880	0.872	0.903	0.841	0.870	0.902	0.824	0.343	0.947	0.885	0.875	0.908	0.833	0.879	0.832	0.916	0.339	0.936
gbdt	0.872	0.856	0.897	0.788	0.872	0.923	0.814	0.356	0.943	0.859	0.840	0.890	0.764	0.856	0.801	0.916	0.376	0.914
lgbm	0.871	0.856	0.897	0.787	0.870	0.925	0.814	0.359	0.943	0.853	0.844	0.882	0.806	0.844	0.789	0.882	0.383	0.928

ACC, accuracy; BA, balanced accuracy; SPE, specificity; PRE, precision; JAC, jacard; MSE, mean square error; AUC, area under the curve; RF, random forest, XGBoost, extreme gradient boosting; CatBoost, categorical boosting; GBDT, gradient boosting decision tree; Lightgbm, light gradient boosting machine

### Visualization analysis of clinical-radiomic CatBoost model

**Fig. 8:**
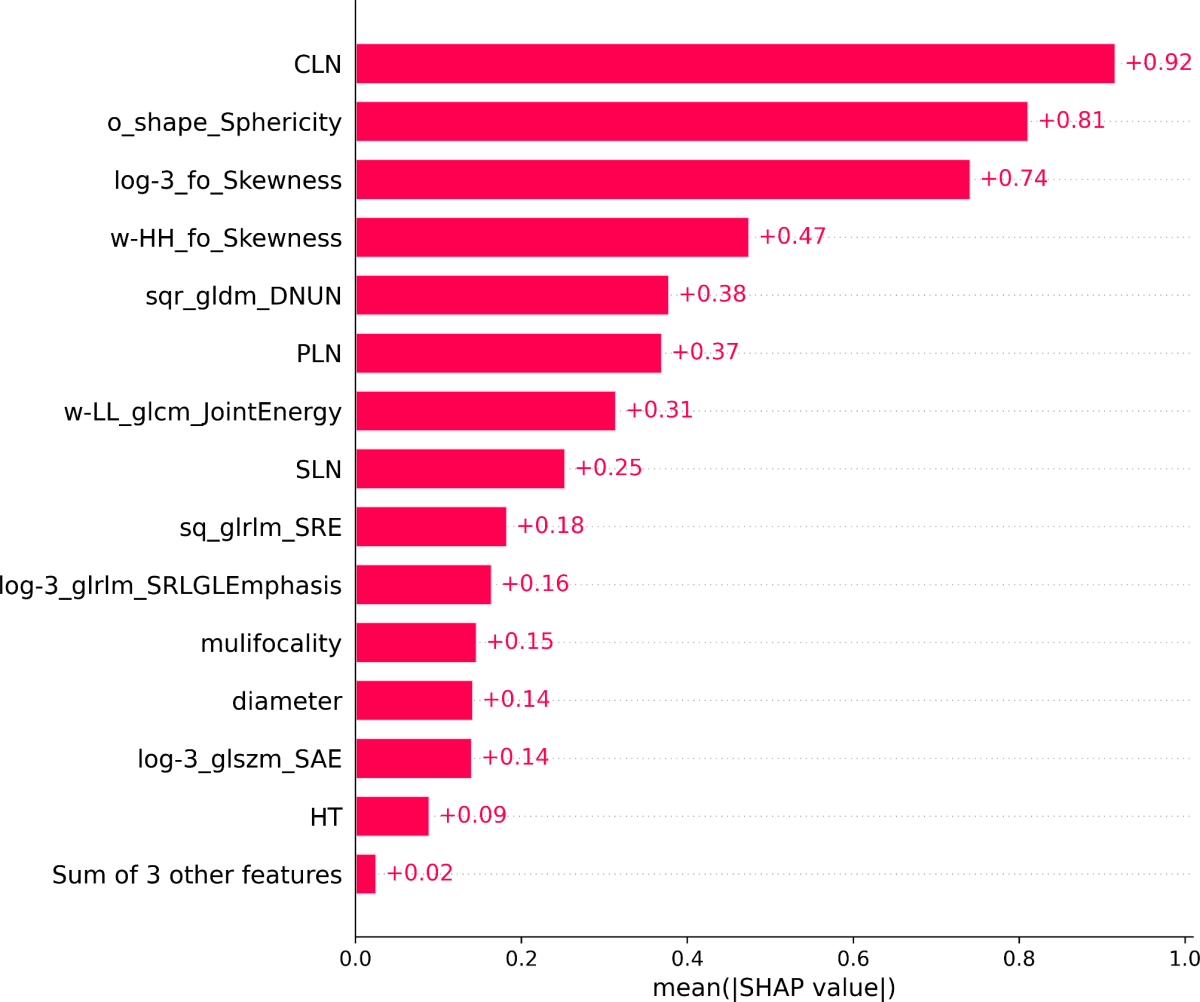
The bar chart in the SHAP summary plot illustrates the impact of each feature on the CatBoost model

**Fig. 9:**
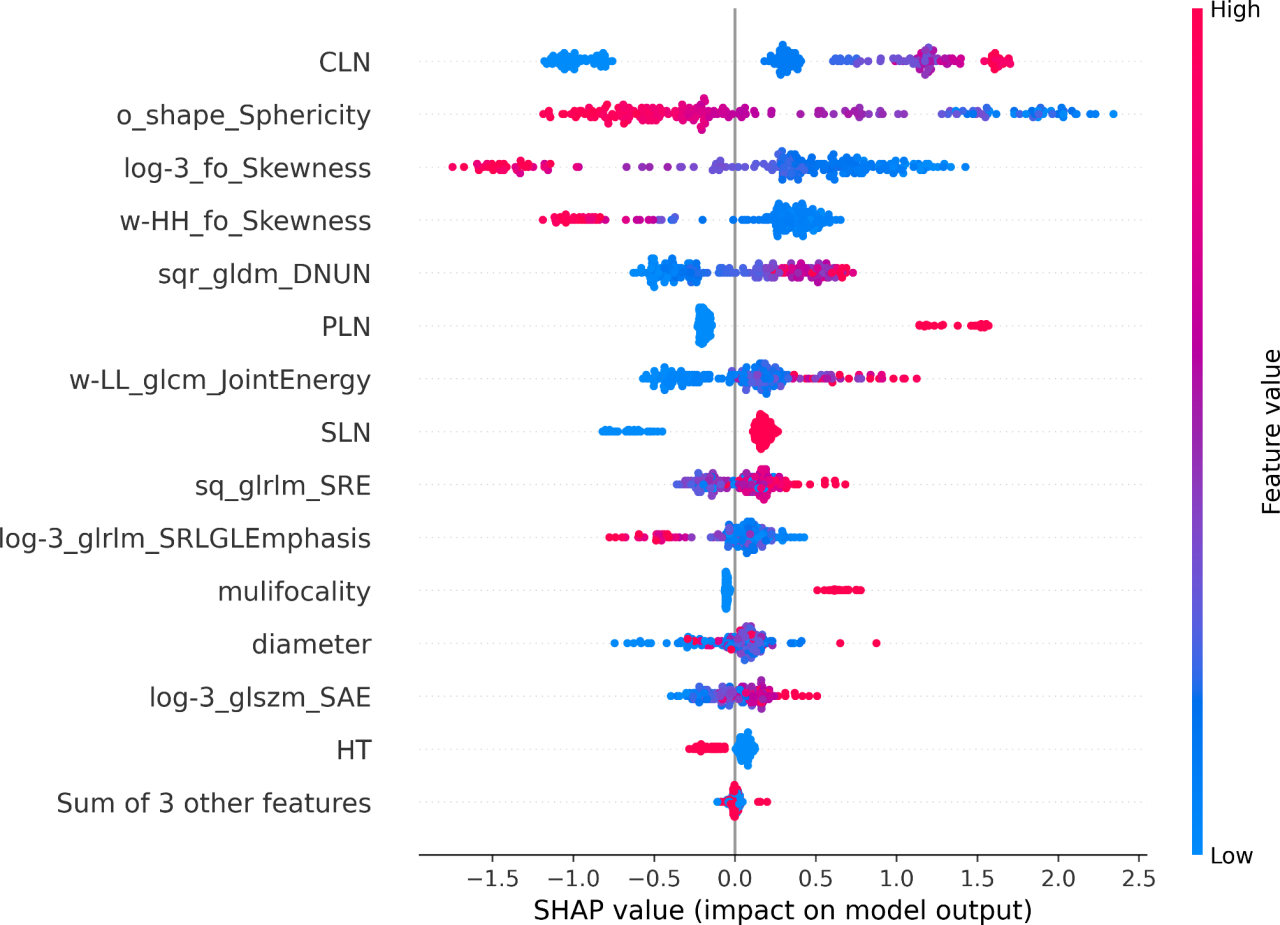
The scatter plot in SHAP summary graph visually depicts the relationship between feature values and predicted probabilities through color

We employ Shapley values to visualize the impact of specific clinical and radiomics features on diagnostic outcomes in a clinical-radiomic CatBoost model. The SHAP bar chart (Fig. 8) illustrates the mean SHAP scores for features, highlighting CLN, o_shap_sphericity, log-3_fo_skewness, w-HH_fo_skewness, and sqr_gldm_DNUN as the most influential factors impacting the outcomes. The SHAP scatter plot (Fig. 9) visually represents the distribution of LLNM and non-LLNM by utilizing red and blue colors to indicate positive or negative effects of features on prediction outcomes. Specifically, o_shap_sphericity, log-3_fo_skewness, and w-HH_fo_skewness exhibit a detrimental impact on the results based on Shapley values, while CLN and sqr_gldm_DNUN demonstrate a favorable influence.

**Fig. 10:**
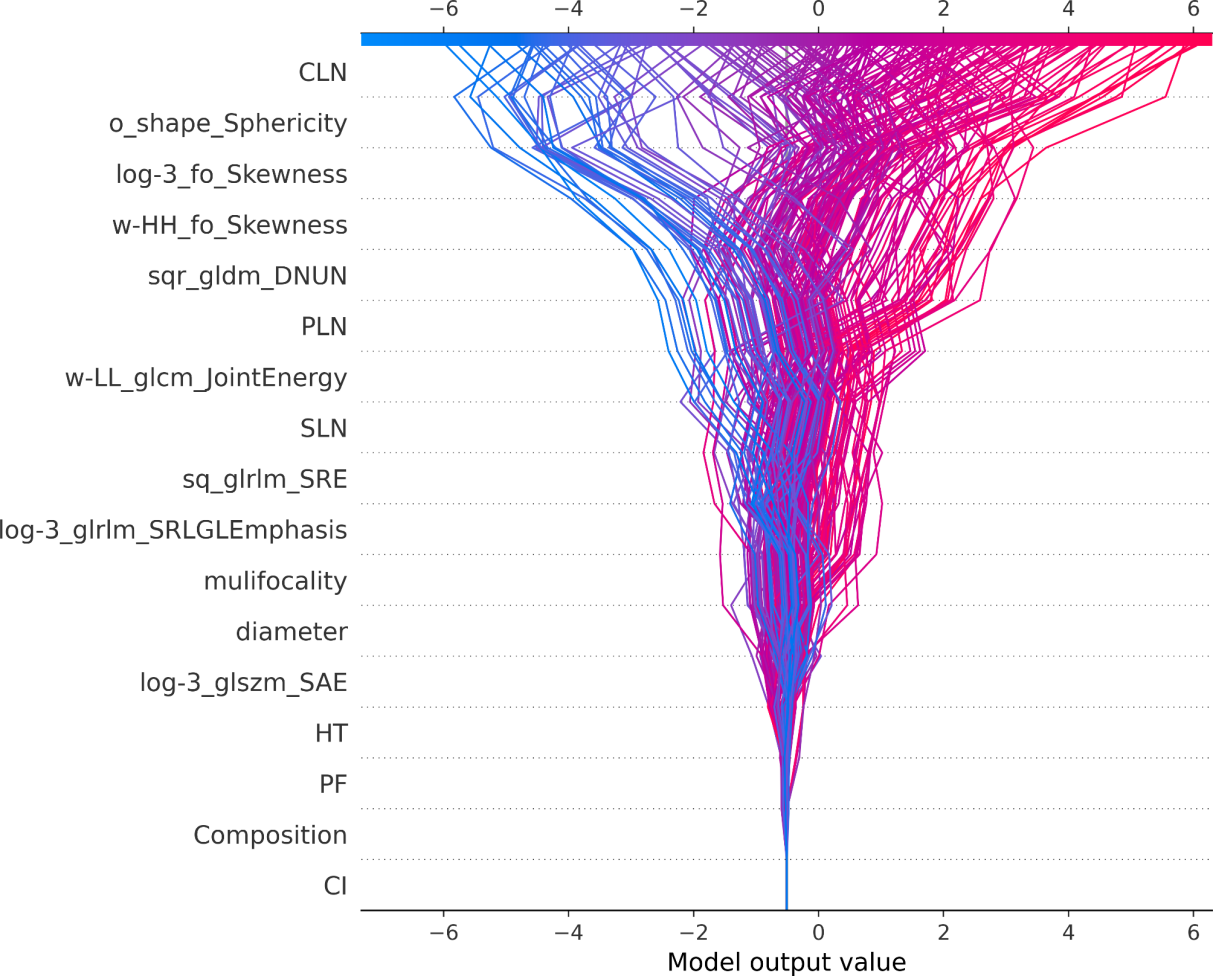
The SHAP decision plot for all patients with PTC

The SHAP decision plot (Fig. 10) visually depicts the impact of each key feature on the final decision, where individual-colored lines represent predicted outcomes for patients. The SHAP values of each element are incrementally added to the baseline of the CatBoost model, illustrating visually how individual features contribute to predictions. Ultimately, at the apex, each line intersects with its corresponding predicted value on the X-axis, representing the final probability prediction of the model.

**Fig. 11:**
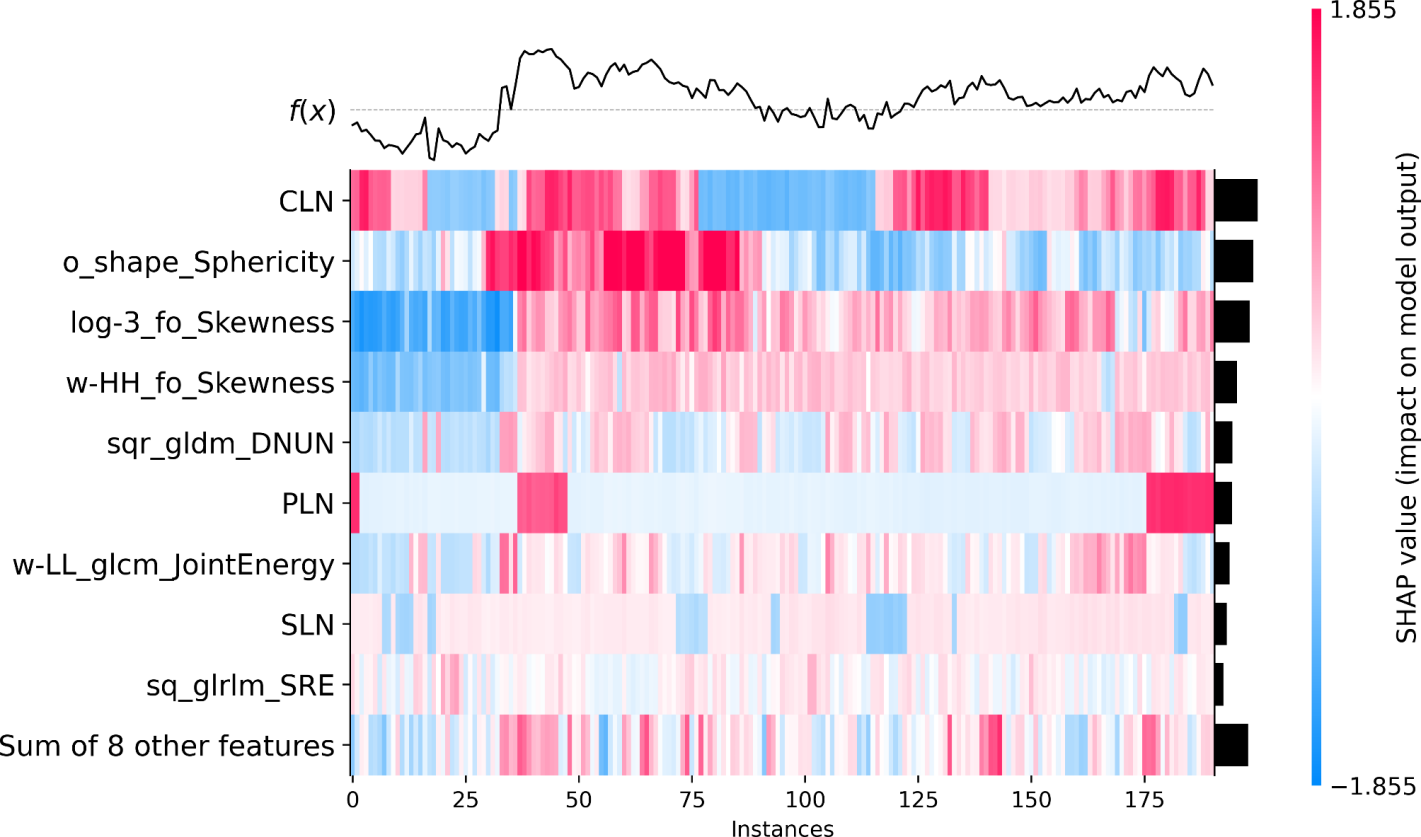
The SHAP heat map effectively visualizes the impact of different features on model output

The SHAP heat map (Fig. 11) visualizes how different features impact the model output. Each row in the heatmap represents a specific feature, while each column presents an individual instance. Each cell’s color represents the SHAP value, which measures the contribution of a feature to the prediction for that instance. A red cell indicates that the feature increases the prediction, while a blue cell indicates that the feature decreases it. A white cell means that the feature has no impact on the prediction. The line graph depicted above the heat map illustrates the values of output f(x) for each individual case. The x-axis is labeled with instance numbers, while the y-axis is scaled based on the output range. Line charts facilitate the visualization of prediction variations across diverse instances and their correlation with the SHAP values of features.

## Discussion

The present study retrospectively analyzed a cohort of 454 patients diagnosed with PTC who had undergone either lateral neck lymph node dissection or intraoperative frozen biopsy. Ensemble learning models have demonstrated outstanding performance in predicting lateral cervical small lymph node metastasis of PTC. Notably, the clinical-radiomic CatBoost model exhibited superior performance with AUC values of 0.95 and 0.94 for the training and testing sets, respectively. The radiomics model exhibited significantly higher AUC values compared to the clinical model in both the training set (0.89 vs. 0.81) and the testing set (0.90 vs. 0.71), within a single model. Compared to a single model, the clinical-radiomic integrated model not only ensures higher sensitivity (0.926 vs. 0.90) based on the radiomics model but also demonstrates improved specificity (0.83 vs. 0.76). The influential parameters affecting the judgment of LLNM were extracted based on this analysis, with the top five parameters identified as CLN, o_shap_sphericity, log-3_fo_skewness, w-HH_fo_skewness and sqr_gldm_DNUN. We employed SHAP to visualize explanations for both individuals and the overall population, where SHAP values represent the significance of features. The red and blue colors indicate positive and negative impacts on the outcome, providing robust evidence for clinical decision-making [20].

Comprehensive clinical studies have been carried out to predict lymph node metastasis based on the clinical features and pathological classification of papillary thyroid carcinoma [22]. Additionally, there exist nomogram and machine learning models for predicting the metastatic status of cervical lymph nodes on the PTC side based on ultrasound characteristics [3, 23–25]. However, there is a scarcity of established models utilizing radiomics features extracted from ultrasound images [9]. Recently, Zhu et al. [26]. developed a clinical-ultrasound nomogram based on multicenter data to predict lateral cervical lymph node metastasis in patients with PTC. In their internal validation set, the AUC of their study was higher than that of our clinical model (0.82 vs. 0.71), but lower than that of the clinical-radiomic CatBoost model (0.82 vs. 0.94). The suboptimal performance of our study’s individual clinical model may be attributed to the relatively small size of our dataset compared to theirs. The incorporation of radiomics features significantly enhances prediction accuracy, potentially capturing underlying tissue heterogeneity in PTC that is imperceptible to the naked eye, such as nodule density and uniformity. These characteristics can be extracted through radiomics analysis and provide valuable evidence for model evaluation [14]. A study conducted by Feng et al. and Lai et al. investigated the utilization of machine learning models for predicting LLNM in PTC based on clinical ultrasound features. The findings demonstrated that the RF model exhibited superior performance, aligning with the outcomes of this investigation [3, 25]. Vivian Y et al. [9] constructed ROC curves by extracting radiomic features such as texture, wavelet, and first-order statistics from ultrasound images of primary tumors. In the validation cohort, the AUC value of this model was significantly lower than that of our clinical-radiomic model in this study (0.62 vs. 0.94). Building upon Vivian Y’s research, we incorporated additional clinical-pathological features into our model, which not only encompassed characteristics of the lesions themselves but also included extranodal features such as CLN status, PLN status, and ultrasound indications of SLN. This integration to some extent improved the performance of our model.

The CatBoost clinical-radiomic model employed in this study successfully identified 17 features through rigorous feature selection techniques and effectively visualized their impact using SHAP. This approach not only accounts for the individual effects of factors on the outcome but also considers the synergistic interactions among different variables [20]. The magnitude of the absolute value of SHAP indicates the influence of features on the results. Our findings reveal that a significant proportion is attributed to expressing grayscale distribution features, including LoG-sigma3_first order_Skewness, LoG-sigma3_glrlm_ShortRunLowGrayLevelEmphasis, wavelet-HH_first order_Skewness, and square_glrlm_ShortRunEmphasis. It can be observed that first-order statistical characteristics of pixel grayscale values and short run gray level intensity have a substantial impact on prediction accuracy, reflecting both overall grayscale distribution patterns and local grayscale intensity conditions. Furthermore, a significant correlation exists between the sphericity of shape features and the predictive outcomes. As the sphericity approaches 1, indicating smoother and more circular primary lesions in PTC, there is a decrease in the likelihood of metastasis. Conversely, when the sphericity is below 0.5 or close to 0, there is an increase in the probability of metastasis. After conducting experimental analysis, this study identified several effective ultrasound image filtering methods for predicting lateral neck lymph node metastasis in PTC, including the original image, wavelet transform, LoG-σ = 3, square transformation, and square root transformation. The radiomics feature, wavelet-HH_first order_Skewness is derived from the application of wavelet transform. The high-pass filtering employed in wavelet transform effectively enhances diagonal edges and image details, as well as provides valuable edge and shape information. In addition to omic features, a plethora of research has demonstrated that CLNM exerts a substantial influence on LLNM, which aligns with the findings of this study. This not only augments the risk of ipsilateral LLNM but also escalates the likelihood of contralateral metastasis [27]. Furthermore, risk stratification will incorporate the quantity of CLNM, with commonly utilized thresholds in clinical practice being 2, 3 and 5 [4, 17, 28]. Currently, a plethora of clinical studies substantiate the pivotal role of CLN status in guiding follow-up and treatment strategies. For patients afflicted with CLNM, specialized examination techniques should be employed to assess the LLN, while regular surveillance suffices for patients without CLNM [29].

The combined CatBoost model can help in the early detection of metastasis, leading to timely and potentially more effective treatment, and it is a non-invasive method which could reduce the need for more invasive procedures like biopsies. Meanwhile by accurately predicting metastasis, the model can aid in tailoring personalized treatment plans for patients. However, this study also has the following limitations. First of all, the data collection is based on a single-center retrospective study, which lacks external verification of the model, and the generalization ability of the model cannot be improved. Meanwhile, ultrasound doctors may have different evaluation criteria for suspicious small lymph nodes, so it is necessary to unify evaluation indicators and conduct prospective multi-center exploration on its practicability. Secondly, our reliance on preoperative fine needle puncture or intraoperative grade III/IV lymph node biopsy as the sole basis for lateral cervical lymph node dissection may lead to an increase in the incidence of false negatives. Finally, while the initial setup might add cost-related efforts, the long-term use of these models can be cost-effective by reducing unnecessary treatments and procedures. At the same time, the software is being developed, and it is reasonable and effective to put into the clinic, and it does not require much cost in future application and maintenance.

## Conclusions

1. This study presents an ensemble learning model to predict the presence of small lymph node metastasis in the lateral cervical of PTC based on clinical and radiomic features.
2. Among these models, the CatBoost model demonstrates superior performance, with its results effectively visualized using SHAP for enhanced interpretability.
3. The combined CatBoost model can improve the diagnostic accuracy of suspicious lymph nodes with short diameter < 8 mm that are difficult to obtain accurate puncture results. The combined application of radiomics features is more accurate and reasonable than the prediction of clinical data alone, which helps to accurately evaluate the surgical scope and provide support for individual clinical decision making.

## Electronic supplementary material

Below is the link to the electronic supplementary material.

Supplementary Information 1

## Data Availability

All data generated or analysed during this study are included in this published article.

## Abbreviations

AUC: Area Under the Curve
BA: Balanced Accuracy
CDFI: Color Doppler Flow Imaging
CLN: Central Lymph Nodes
CLNM: Central Lymph Node Metastasis
CT: Computed Tomography
DET: Detection Error Tradeoff
FNA-Tg: Fine Needle Aspiration Thyroglobulin
GBDT: Gradient Boosting Decision Tree
RF: Random Forest
XGBoost: Extreme Gradient Boosting
CatBoost: Categorical Boosting
Lightgbm: Light Gradient Boosting Machine
LLNM: Lateral Lymph Node Metastasis
MRI: Magnetic Resonance Imaging
MSE: Mean Square Error
NLR: Neutrophil Lymphocyte Ratio
PLN: Prelaryngeal Lymph Node
PLR: Platelet to Lymphocyte ratio
PTC: Papillary Thyroid Carcinoma
PTMC: Papillary Thyroid Microcarcinoma
RF: Random Forest
RFE: Recursive Feature Elimination
ROI: Region of Interest
SHAP: Shapley Additive exPlanations
SLN: Suspicious Lymph Node
Tg: Thyroglobulin
TgAb: Thyroglobulin Antibodies
TPO-Ab: Thyroid Peroxidase Antibody
TSHR: Thyroid-stimulating Hormone Receptor

## References

[1] Megwalu UC, Moon PK. Thyroid Cancer Incidence and Mortality trends in the United States: 2000–2018. Thyroid. 2022;32:560–70. 10.1089/thy.2021.0662 35132899 10.1089/thy.2021.0662

[2] So YK, Kim MJ, Kim S, Son YI. Lateral lymph node metastasis in papillary thyroid carcinoma: a systematic review and meta-analysis for prevalence, risk factors, and location. Int J Surg. 2018;50:94–103. 10.1016/j.ijsu.2017.12.029 29329789 10.1016/j.ijsu.2017.12.029

[3] Lai SW, et al. Machine learning-based dynamic prediction of lateral lymph node metastasis in patients with papillary thyroid cancer. Front Endocrinol (Lausanne). 2022;13:1019037. 10.3389/fendo.2022.1019037 36299455 10.3389/fendo.2022.1019037 PMC9589512

[4] Hu D, et al. Risk factors of lateral lymph node metastasis in cN0 papillary thyroid carcinoma. World J Surg Oncol. 2018;16. 10.1186/s12957-018-1336-3 10.1186/s12957-018-1336-3 PMC5811970 29439716

[5] Schneider DF, Mazeh H, Chen H, Sippel RS. Lymph node ratio predicts recurrence in papillary thyroid cancer. Oncologist. 2013;18:157–62. 10.1634/theoncologist.2012-0240 23345543 10.1634/theoncologist.2012-0240 PMC3579599

[6] Gao L, et al. Large-volume lateral lymph Node Metastasis predicts worse prognosis in papillary thyroid carcinoma patients with N1b. Front Endocrinol (Lausanne). 2021;12:815207. 10.3389/fendo.2021.815207 35185788 10.3389/fendo.2021.815207 PMC8847215

[7] Huang K, Ji-Bin, Liu. Application of Ultrasonography in the diagnosis and management of papillary thyroid microcarcinoma. Adv Ultrasound Diagnosis Therapy. 2020;4(4):284–90. 10.37015/AUDT.2020.200001

[8] Lee DW, et al. Roles of ultrasonography and computed tomography in the surgical management of cervical lymph node metastases in papillary thyroid carcinoma. Eur J Surg Oncol. 2013;39:191–6. 10.1016/j.ejso.2012.07.119 22863305 10.1016/j.ejso.2012.07.119

[9] Park VY, et al. Radiomics signature for prediction of lateral lymph node metastasis in conventional papillary thyroid carcinoma. PLoS ONE. 2020;15:e0227315. 10.1371/journal.pone.0227315 31940386 10.1371/journal.pone.0227315 PMC6961896

[10] Perros P, et al. Guidelines for the management of thyroid cancer. Clin Endocrinol (Oxf). 2014;81(Suppl 1):1–122. 10.1111/cen.12515 24989897 10.1111/cen.12515

[11] Haugen BR, et al. American Thyroid Association Management Guidelines for adult patients with thyroid nodules and differentiated thyroid Cancer: the American Thyroid Association Guidelines Task Force on thyroid nodules and differentiated thyroid Cancer. Thyroid. 2015;26:1–133. 10.1089/thy.2015.0020. (2016). 10.1089/thy.2015.0020 PMC4739132 26462967

[12] Leenhardt L et al. 2013 European thyroid association guidelines for cervical ultrasound scan and ultrasound-guided techniques in the postoperative management of patients with thyroid cancer. *Eur Thyroid J* 2, 147–159, 10.1159/000354537 (2013). 10.1159/000354537 PMC4017749 24847448

[13] Xia S, Chen Y, Zhan W, Zhou W. Ultrasound-guided fine-needle aspiration Versus Fine-Needle Capillary Sampling in evaluation of Lymph Node metastasis of thyroid Cancer. Front Oncol. 2021;11:642142. 10.3389/fonc.2021.642142 33937044 10.3389/fonc.2021.642142 PMC8079778

[14] Agyekum EA, et al. Evaluation of Cervical Lymph Node Metastasis in papillary thyroid carcinoma using clinical-Ultrasound Radiomic Machine Learning-based model. Cancers (Basel). 2022;14. 10.3390/cancers14215266 10.3390/cancers14215266 PMC9655605 36358685

[15] Zhu H, et al. Models of ultrasonic radiomics and clinical characters for lymph node metastasis assessment in thyroid cancer: a retrospective study. PeerJ. 2023;11:e14546. 10.7717/peerj.14546 36650830 10.7717/peerj.14546 PMC9840861

[16] Xiao L, et al. Contrast-enhanced US with Perfluorobutane to diagnose small lateral cervical lymph node metastases of papillary thyroid carcinoma. Radiology. 2023;307:e221465. 10.1148/radiol.221465 37014242 10.1148/radiol.221465

[17] Huang Y, Yin Y, Zhou W. Risk factors for central and lateral lymph node metastases in patients with papillary thyroid Micro-carcinoma: retrospective analysis on 484 cases. Front Endocrinol (Lausanne). 2021;12:640565. 10.3389/fendo.2021.640565 33746905 10.3389/fendo.2021.640565 PMC7973362

[18] Shao L, Wang Z, Dong W, Sun W, Zhang H. Risk factors associated with preferential lateral lymph node metastasis in papillary thyroid carcinoma. Cancer Med. 2023;12:20670–6. 10.1002/cam4.6567 37905599 10.1002/cam4.6567 PMC10709716

[19] Zhao M, et al. Predicting skip metastasis in lateral lymph nodes of papillary thyroid carcinoma based on clinical and ultrasound features. Front Endocrinol. 2023;14:ARTN. 10.3389/fendo.2023.1151505 10.3389/fendo.2023.1151505 PMC10203516 37229457

[20] Zhang G, et al. A machine learning model based on ultrasound image features to assess the risk of sentinel lymph node metastasis in breast cancer patients: applications of scikit-learn and SHAP. Front Oncol. 2022;12:944569. 10.3389/fonc.2022.944569 35957890 10.3389/fonc.2022.944569 PMC9359803

[21] Bai BL, Wu ZY, Weng SJ, Yang Q. Application of interpretable machine learning algorithms to predict distant metastasis in osteosarcoma. Cancer Med. 2023;12:5025–34. 10.1002/cam4.5225 36082478 10.1002/cam4.5225 PMC9972029

[22] Medas F, et al. Predictive Factors of Lymph Node Metastasis in patients with Papillary Microcarcinoma of the thyroid: retrospective analysis on 293 cases. Front Endocrinol (Lausanne). 2020;11:551. 10.3389/fendo.2020.00551 32982963 10.3389/fendo.2020.00551 PMC7477034

[23] Feng JW, et al. Nomograms for prediction of high-volume lymph node metastasis in papillary thyroid carcinoma patients. Otolaryngol Head Neck Surg. 2023;168:1054–66. 10.1002/ohn.161 36856043 10.1002/ohn.161

[24] Wang Y, et al. Risk factors and a prediction model of lateral lymph node metastasis in CN0 papillary thyroid carcinoma patients with 1–2 Central Lymph Node metastases. Front Endocrinol (Lausanne). 2021;12:716728. 10.3389/fendo.2021.716728 34721289 10.3389/fendo.2021.716728 PMC8555630

[25] Feng JW, et al. A comparative analysis of eight machine learning models for the prediction of lateral lymph node metastasis in patients with papillary thyroid carcinoma. Front Endocrinol (Lausanne). 2022;13:1004913. 10.3389/fendo.2022.1004913 36387877 10.3389/fendo.2022.1004913 PMC9651942

[26] Zhu J, et al. Nomogram for preoperative estimation risk of lateral cervical lymph node metastasis in papillary thyroid carcinoma: a multicenter study. Cancer Imaging. 2023;23:55. 10.1186/s40644-023-00568-5 37264400 10.1186/s40644-023-00568-5 PMC10236734

[27] Machens A, Hauptmann S, Dralle H. Lymph node dissection in the lateral neck for completion in central node-positive papillary thyroid cancer. Surgery. 2009;145:176–81. 10.1016/j.surg.2008.09.003 19167972 10.1016/j.surg.2008.09.003

[28] Liu C, et al. Risk factor analysis for predicting cervical lymph node metastasis in papillary thyroid carcinoma: a study of 966 patients. BMC Cancer. 2019;19:622. 10.1186/s12885-019-5835-6 31238891 10.1186/s12885-019-5835-6 PMC6593593

[29] Lan X, et al. A Meta-analysis of Central Lymph Node Metastasis for Predicting lateral involvement in papillary thyroid carcinoma. Otolaryngol Head Neck Surg. 2015;153:731–8. 10.1177/0194599815601412 26307575 10.1177/0194599815601412

