# Supplementary material for: Prediction of lateral lymph node metastasis with short diameter less than 8 mm in papillary thyroid carcinoma based on radiomics
Source: Cancer Imaging. 2024 Nov 15;24:155. doi: 10.1186/s40644-024-00803-7 (PMC11566407; doi:10.1186/s40644-024-00803-7)
Supplement: Supplementary file 1 — Supplementary Information 1 [file 40644_2024_803_MOESM1_ESM.docx]

| clinical features | omics feasures | clinical-omics feasures |
| --- | --- | --- |
| diameter | o_shape_Sphericity | o_shape_Sphericity |
| mulifocality | log-3_fo_Skewness | log-3_fo_Skewness |
| Composition | log-3_glrlm_SRLGLEmphasis | log-3_glrlm_SRLGLEmphasis |
| posterior features（PF） | w-HH_fo_Skewness | w-HH_fo_Skewness |
| SLN | w-LL_glcm_JointEnergy | w-LL_glcm_jointEnergy |
| PLN | sqr_gldm_DNUN | sqr_gldm_DNUN |
| CLN | log-3_glszm_SAE | log-3_glszm_SAE |
| HT | sq_glrlm_SRE | sq_glrlm_SRE |
| Capsular invasion（CI） |  | diameter |
| age |  | mulifocality |
|  |  | Composition |
|  |  | PF |
|  |  | SLN |
|  |  | PLN |
|  |  | CLN |
|  |  | HT |
|  |  | CI |
|  |  |  |

SLN, suspicious lymph nodes; PLN, prelaryngeal lymph node; CLN, central lymph node; HT, hashimoto's thyroiditis;
